# Investigating the disjoint between education and health policy for infant feeding among teenage mothers in South Africa: a case for intersectoral work

**DOI:** 10.1186/s12889-021-12435-8

**Published:** 2022-01-06

**Authors:** Jo Hunter-Adams, Anna Strebel, Joanne Corrigall, Virginia Zweigenthal

**Affiliations:** 1 Health Economics Unit, School of Public Health and Family Medicine, Faculty of Health Sciences, University of Cape Town, Observatory, Cape Town, 7925 South Africa; 2 Women’s and Gender Studies, Faculty of Arts, University of the Western Cape, Bellville, 7535 South Africa; 3 Primary Health Care Directorate, Faculty of Health Sciences, University of Cape Town, Observatory, Cape Town, 7925 South Africa; 4 Division of Public Health Medicine, School of Public Health and Family Medicine, Faculty of Health Sciences, University of Cape Town, Observatory, Cape Town, 7925 South Africa

**Keywords:** *Breastfeeding*, Infant formula, Pregnancy in adolescence, Health policy, women’s health

## Abstract

**Background:**

Many low-and-middle-income countries, including South Africa, have high rates of teenage pregnancy. Following the World Health Organisation recommendations, South African health policy on infant feeding promotes exclusive breastfeeding until six months of age, with gradual weaning. At the same time, South Africa’s education department, in the interest of learners, promotes adolescents’ early return to school post-partum. Yet infant feeding at school is currently not perceived as a realistic option.

**Methods:**

Recognising his this policy tension, we aimed to explore how policies are interpreted and implemented by the health and education sectors through interviews with key informants who produce, interpret and implement these policies. Using an interview guide developed for this study, we conducted in-depth interviews with 24 health policy makers, managers in both sectors, school principals and nursing staff who manage adolescent mothers (aged 16-19) and their babies. Data was analysed using thematic analysis.

**Results:**

Informants from both sectors expressed discomfort at pregnant learners remaining in school late in pregnancy and were uncertain about policy regarding when to return to school and how long to breast-feed. Educators reported that new mothers typically returned to school within a fortnight after delivery and that breastfeeding was not common. While health professionals highlighted the benefits of extended breastfeeding for infants and mothers, they recognised the potential conflict between the need for the mother to return to school and the recommendation for longer breastfeeding. Additionally, the need for ongoing support of young mothers and their families was highlighted.

**Conclusions:**

Our findings suggest educators should actively encourage school attendance in a healthy pregnant adolescent until delivery with later return to school, and health providers should focus attention on breastfeeding for the initial 4-6 weeks postpartum, followed by guided support of formula-feeding. We encourage the active engagement of adolescents’ mothers and extended families who are often involved in infant feeding and care decisions. Education and health departments must engage to facilitate the interests of both the mother and infant: some exclusive infant feeding together with a supported return to school for the adolescent mother.

**Supplementary Information:**

The online version contains supplementary material available at 10.1186/s12889-021-12435-8.

## Background

The ‘first 1000 days of life’ – the time from conception to a child’s second birthday – is the critical window of opportunity for the optimal development of children [1]. This perspective acknowledges that country policies, social contexts and interventions that promote the well-being of mothers, also promote child health [2]. Infant feeding practices impact on the health and wellbeing of both infants and mothers.

Following WHO and UNICEF recommendations [3], in South Africa (SA), exclusive breastfeeding is advocated until six months of age, with gradual weaning [4], regardless of HIV status and age. Despite this policy, breastfeeding rates in SA are low among HIV positive and negative women of all ages [5, 6].

Although there are initiatives to reduce teenage pregnancy, such as postponing sexual debut [7], SA has high levels of teenage pregnancy. In 2017, 109 births per 1000 were born to women aged 10-19 [8]. Globally, an estimated 16 million adolescents give birth each year. Of these adolescents, 95% live in low-and-middle income countries [9].

Adolescent antenatal care requires special attention, to ensure the health of both mother and infant, as pregnancy in adolescence is associated with greater health risk to the mother and newborn — including anaemia, mortality, stillbirths and prematurity [10]. Babies of adolescents face the highest risk of infant and child mortality, as well as other health problems, such as stunting, diarrhoea and anaemia [11].

Child-bearing often prevents adolescent mothers from attending school. In LMICs, adolescent pregnancy is a severe impediment to development, and can lead to a number of challenges, including abandonment by their partners, school dropout and lost productivity, which ultimately limits young women’s future social and economic opportunities, leading to intergenerational transmission of poverty [12]. SA current policy intends to facilitate young mothers’ continued learning, encouraging a return to school to complete secondary school education [13].

As found in the United States, SA studies show that breastfeeding amongst adolescent mothers is low [14], and a recent qualitative study showed that adolescent mothers who return to school are less likely to exclusively breastfeed [15]. Other SA studies have reported that mothers introduced complementary foods or fluids earlier than recommended if returning to work or school [16, 17].

There is a need to develop health policy and implement proven interventions promoting good infant nutrition in LMIC settings with a higher burden of undernutrition and food insecurity [18]. The WHO advocates for women in “difficult circumstances” to be offered “practical support” for feeding options, preferably breastfeeding. This includes adolescents, in addition to malnourished women and infants, and HIV-infected women where diarrhoea and malnutrition are prevalent [19].

Our previous research with adolescent women, which included adolescent mothers, living in low-socioeconomic peri-urban areas of Cape Town, SA about perceptions and experiences of infant feeding found that they appreciated the need to complete schooling, but felt that this largely precluded exclusively breastfeeding. Yet they understood the advantages of breastfeeding [2]. This accords with SA’s educational policies that are framed to advance the interests of the pregnant adolescent – namely to return to school to complete schooling as soon as possible [13], as a delay may result in dropping out of school. On the other hand, health policy is framed to advance the interest of the child – to be exclusively breast-fed for six months, thus promoting HIV-free survival and optimal growth. These policies were developed in silos but need to speak to the comprehensive needs of adolescent mothers and their infants.

Our follow-up study, reported here, among policy makers and implementers in the education and health sectors in the Western Cape Province in SA, explored the implementation of seemingly contradictory health policies that promote exclusive breastfeeding and education policies that promote the completion of schooling for young women post-partum, and their recommendations. School attendance prior to delivery affects the length of time adolescent mothers are absent from school. Absenteeism impacts on school completion and consequently, school attendance by pregnant learners was explored with informants.

## Methods

We conducted a qualitative study to explore the implementation of the ‘exclusive breastfeeding to six months’ and ‘return to school’ policies among provincial policy-makers and implementers, and academics supporting health and education services. Through semi-structured interviews, which are appropriate to explore the complex phenomena in this study, we explored the seeming contradiction of health and educational policy with key informants, obstacles and facilitators of their implementation, and ways that contradictions can be overcome. The interview guide used is included in the supplementary material. The 24 stakeholders interviewed through 18 interviews, by sector, are given in Table 1.

**Table 1:** Informants interviewed by sector

Health Sector		Education Sector	
Informants	number	Informants	number
Programme managers	5	Provincial officials	6
Nursing staff	4	School principals	4
Specialist physicians	1	Supporting institutions	4

We carried out purposive sampling, identifying key stakeholders who could provide rich information about the history of policies, their current formulation and implementation. These included provincial health and education managers, health researchers as well as facility - clinic and high school principals/managers, and frontline staff (clinic nurses and teachers). Facility informants were drawn from four schools and four clinics; two in the areas where we had conducted our infant feeding research among adolescents and two from areas which reported high numbers of adolescent pregnancies. Potential informants were emailed to inform them about the study, and interviews were then arranged telephonically. After written consent for participation and audio-recording that assured informants’ anonymity, all interviews were conducted in-person in English. All were one-on-one, except an education interview with six informants and one health interview with two programme managers, responsible for policy around nutrition and the prevention of transmission of HIV infection from mothers to children. Field notes were taken by the one researcher who conducted all interviews. All recordings were transcribed verbatim by one professional transcriber. Data were analyzed by hand inductively, following immersion in the material, identifying key themes, relationships between themes and identifying quotations that best described the varied knowledge, perceptions and experiences of informants. Ethics approval was obtained from the University of Cape Town’s Human Research Ethics Committee (Ref 416/2018).

## Results

The main issues that emerged from the interviews with key informants in both the education and health sectors centred on discussion about how long the pregnant learner should remain in school; when the young mother should return to school; perceptions of how adolescent mothers experienced and approached breastfeeding; challenges regarding breastfeeding at school and in policy; as well as support for breastfeeding.

### School before birth

Especially among educators, there was discussion about the management of pregnant learners in the school context. Some informants indicated that a Learner Pregnancy Policy required that pregnant learners needed to obtain a medical certificate about their condition, regarding how long to stay in the classroom before giving birth, which was determined in discussion with the learner and her parent/s:

*if the learner finds out that she’s pregnant, then it is requested that the learner must produce a medical certificate to say whether it’s advisable to remain at school or withdraw from school. I don’t think the policy says much about timing. But what it does say is that … the decision-making must be taken by professionals. And there should be guidance from the psychological services, psychologists. The principal has to get involved and … so the policy I don’t think is very prescriptive on time [coughs] Because every situation is relative. Right? [Education]*

*We set up a meeting with them. We discuss how we’re going to handle the pregnancy, till what time will they come to school, and then when would they be coming back to school … [Education]*

There was a sentiment, also shared by some in the health sector, that pregnant learners should not stay at school too far into the pregnancy, as the school could not ensure their safety, deal with any problems that might arise in the pregnancy, or a delivery on school grounds. School work could then be supplied for her to work on at home, in the form of learning materials, or even Saturday classes:

*They don’t usually write exams with other learners, especially if they’re close to the time, … because what if they give birth, know what I mean, at school, which would be a very tricky situation. Because we don’t have nurses and some sort of backup for that type of thing. So a lot of the time, they’re at home just before. When I say just before, I mean maybe a month before. [Education]*

*So we shouldn’t discriminate against a learner who becomes pregnant. We should try and work, so that a solution is found, so that they can continue their schooling. And we can just … be accommodating in that regard. I also believe that … the learner can attend school … as late into the pregnancy as possible. I personally am very scared about that, because I think after seven months, anything can happen. And that would actually be to the disadvantage of the learner, because we’re not trained to deal with any emergencies. [Education]*

*They get supported by the school with … an academic information package, or whatever’s being done in class textbook-wise. [Education]*

### When to return to school?

A central point of discussion for many informants was when the learner should return to school after delivery. There were a range of opinions among educators about what the Department of Education’s policy was. Some were uncertain as to what the policy was or thought that principals did not know what the policy was. Others believed there was no explicit policy about this:

*Principals, or the people that manages the school, sometimes … don’t know the content of … the Learner Pregnancy Policy. [Education]*

*I don’t know if schools actually chase kids away [laughs] but we don’t have that policy, we accepted her back to start because you know, they … deserve a chance, they deserve … some relief. [Education]*

In practice, some education informants indicated that girls returned to school very soon after they had given birth, as early as one or two weeks. In many instances the timing of return to school was decided at a meeting with the principal, learner and her parents. In some cases, principals were strong advocates of an early return to school. In other cases, the early return was the result of pressure from the family, who would then look after the baby, so that the learner would not lose too much school work, and this would make it easier for her to adjust to school again:

*It’s actually as soon as you can come back. And the parents, I suppose the families, advocate for that because they don’t want them to miss school. … Just on my side, I don’t know of children who stay away more than a week, maybe two weeks, after they gave birth. [Education]*

*And most of the time it’s the mother that will just take the baby as her own child, and let the young mother be free. It’s like the grandmother. So the young mother is free to be a learner at the school again. [Education]*

On the other hand, most informants from both sectors suggested that learners should stay home for longer. They identified a minimum of four to six weeks for breastfeeding, or three to six months and longer, so that they had time to recover:

*So in my opinion, I think the six-week rule should apply for school, for the learner … But concessions should be made for that learner to receive the work and not to fall too far behind with schoolwork, so that she’s able to catch up. [Health]*

*OK, so three months is a really good time, because I know it flies by very quickly. And you need to connect with your child … because it means life-long relationship attachment. And without that there are consequences for that child in the future ... It would be nice to get a three-month sort of timeframe. But I don’t think with the schooling context, that might be possible. But I would still say at least a month. [Education]*

*I, myself, advise the parents to allow the learner to at least take care of her child for up to six months, one year, then come back to school. [Education]*

A strong sentiment among educators was that it should be up to the learner and her family to decide when it was appropriate for her to return to school. Cases needed to be considered individually, and flexibility was needed:

*The family made the decision they needed to go back to school, whether they [the adolescent mother] wanted to or not. So they had to get back for the start of school term. The longest was two weeks before the school term restarted. And the child would’ve gone onto formula. [Education]*

*I see a danger if we prescribe a timeframe. I think each case must be judged on its merits. I know that we are very different. [… For] a particular learner maybe a week might be sufficient. [Education]*

*Yes, I would say that it’s possible to go back to school and to continue breastfeeding. But you must understand our main core mandate as the Department of Education is to provide education to the learner. That is our constitutional mandate. But our policy is also very flexible so caters for situations where the female learner wants to continue breastfeeding. Such a request may be made to the school. [Education]*

### Approaches to breastfeeding for the learner

There was wide-ranging discussion among informants regarding adolescent mothers’ approach to and experience of feeding their infants. Some informants felt that young mothers enjoyed breastfeeding, although many thought that they did not, and also did not understand the importance of breastfeeding:

*And your mention about breastfeeding, with these ones I knew fairly intimately, about five, they all wanted to breast-feed, actually. And they all did, and it was a very precious thing that they did. And even though … they only managed to breastfeed for maybe a week or two, but from a health perspective, even a week or two is fantastic [Education]*

*There’s a negativity about it [breastfeeding] [Health]*

Others thought that these mothers were concerned about their breasts leaking and sagging, that they found breastfeeding to be time-consuming, and that if they breastfed they would be judged by their friends:

*They also don’t want to have the leaking breasts at school ... I suppose you’re still a teenager at the end of the day, and it’s not something that’s talked about or supported enough to be cool. [laughs] [Education]*

*I think, if I give my own opinion, … breastfeeding is time-consuming. You constantly have to sit with this child. And they want to be on the go, they want to go there, and do this story and interact. [Health]*

*You will find, sometimes you will find they are saying, “I can’t breastfeed, my breasts are so painful’, or “I can’t breastfeed, my nipples are cracked’. They come up with stories. Then we check them, let them sit down ... You will find that the baby is latching very well, so what were you saying? No, it is just that I don’t like breastfeeding. So that is why, my friends told me to say this. [Health]*

There was a strong sentiment among some informants that breastfeeding was the right thing to do for young mothers; that it was healthier for both the infant and the mother, that it was cheaper, and that it promoted bonding between mother and infant:

*I think they need to breastfeed. Breastmilk is very important to the baby ... There is a big difference between the baby that is breastfed and the baby that is getting formula milk. [Health]*

*I also read that breastfeeding is much better for children to make them stronger, and their immune systems are much stronger than bottle-fed baby. So in principle, … it is better for a mother to breastfeed her child. [Education]*

*… but also the bonding, the social benefits that goes with breastfeeding. So they clearly don’t understand the interactions that happens during breastfeeding, the act of feeding at the breast, or the act of providing breastmilk to that child. [Health]*

Among educators the sense was that learners preferred to formula-feed their babies:

*Definitely formula-feeding. Very few breastfeed. [Education]*

*So it normally takes a week … They tell me that during that week, they just don’t breast-feed so that it gives them that time to allow the breasts to dry. So that’s what they do. [Education]*

Moreover, there was pressure from friends and particularly their families to formula-feed, especially if they wanted to return to school. Thus, even if the mother was breastfeeding, the parents or extended family who cared for the infant during the day would use formula, with the result that mixed feeding was common:

*The family made the decision they needed to go back to school whether they wanted to or not. And so they had to get back for the start of school term. So the longest was two weeks before the school term restarted and the child would’ve gone onto formula. [Education]*

*Some are at school. So during the day, whoever is staying with the baby will give the baby milk formula. And then she will come back and breastfeed. [Health]*

*Even if they express, but you will find the mother or grand-mama gave something else at home [Health]*

A further consideration was that although it was preferable for these girls to breastfeed, expressing milk was not widely known about, or common in local communities:

*To be honest with you, the idea of expressing is not a very well-known idea. I know it should be, but it’s actually not … And a teenager and her parents might not even know that you can express. Or they know but it’s … a bit strange. [Education]*

While HIV was not raised as a key concern in discussions around breastfeeding, some health informants highlighted research into the value of exclusive breastfeeding for HIV positive mothers, together with adherence to ARV therapy. There was consensus that six months was the recommended time period for breastfeeding in the context of HIV. It, however, needed to be normalised, especially as some young HIV positive mothers were scared to breastfeed. There was also confusion about the risk posed by mixed-feeding in the context of HIV-infection:

*The holy grail is six months exclusive breastfeeding, no doubt about it. But realities dictate that some mixed-feeding happens. Mixed-feeding is better than exclusive formula-feeding, you know, because the benefits of breastfeeding are dose related. [Health]*

*South Africa subscribes to WHO guidelines of six months exclusive breastfeeding. First six months exclusive breastfeeding, and thereafter continue breastfeeding while introducing a mixed diet for two years or more. That now is the same in the HIV context, except with the added bit that the moms need to be adhering to their ARV. [Health]*

*In most cases, they’ve just recently found out that they’re HIV positive, so then that becomes a problem. So they actually need more counselling to teach them … more about HIV and breastfeeding. [Health]*

### Support for breastfeeding adolescent mothers

Given the many challenges, there was recognition of the need for support for breastfeeding with these adolescent mothers. The health department supported young mothers breastfeeding by teaching them how to express milk while they were still in hospital/clinic, and by having breastfeeding counsellors at some facilities. However, once the mother was discharged it was difficult to monitor their progress with feeding. The perception was that family influenced their choices about ongoing feeding:

*They leave the health facility being fully equipped to breastfeed, with knowledge and skill, but they come back into the system having changed the feeding options … When we ask them why, it’s the influence, from the grannies, or the aunties, or the neighbours, or whoever is an influence on that child’s life. [Health]*

*In most cases … you’re giving them health education here at the clinic and there is a breastfeeding counsellor that is educating them. They will understand everything … When they go home then the parents are telling them something different. [Health]*

Suggestions to manage adherence to breastfeeding included home visits by breastfeeding counsellors and establishing support groups. Additionally, it was stressed that the extended family, like the learner’s mother and grandmother, who are often involved in childcare – especially once the new mother returns to school – should be involved in follow-up support around breastfeeding. Informants thought that a group approach would be best:

*The other avenue of support is … where we link the mother either to community-based services, or to a support group in the community. We employ breastfeeding counsellors who run support groups in the community. So we’ll link them with those. [Health]*

*If they can come with the mummy, the grandma. If they can come with the grandma to the clinic so that they can also be educated on the importance of exclusively breastfeeding … So they can also go to the breastfeeding counsellors specifically for that health education. [Health]*

Suggestions were made to use technology like a ‘WhatsApp’ group and videos to provide ongoing support. Some, however, recognized that this might not be financially feasible:

*We do have WhatsApp systems where some of the teachers are on WhatsApp with the learner … Via WhatsApp she can … pose those questions to the educator and get some answers for that. [Education]*

*They can’t really access WhatsApp easily, and on top of that, they can’t afford the data costs. [Health]*

In addition to issues around breastfeeding at school, there were considerations about additional support for mother-learners. Some informants, especially educators, highlighted the need for academic support for the new mother, starting while she is at home after delivery. A designated friend could also bring schoolwork home for her. However, once back at school, the new mother would need extra classes to catch up what she’d missed while at home:

*There could be, you know, a support structure at the peer level … I’m sure some of the girls in the class would be interested in seeing the baby. They could bring the schoolwork and they can chat around the baby, and chat around schoolwork. [Health]*

*What prudent schools do is … they provide lost education during the examination period, or … post-delivery … When the child comes back, they provide that lost tuition, that lost assessment. [Education]*

A further concern found among all informants was that new mothers could experience emotional and adjustment problems. Some thought that issues like post-natal depression were quite common. However, there was generally little effort to identify and manage such issues:

*But a big thing is around mental health and psychological support. I think the Health Department really can do a lot, because she would be very vulnerable. We know that the rate of post-partum depression is even higher in teenagers. Be very aware of potential depression, mental health issues that could occur, screening them … early, making sure that there’s access to mental health support. [Health]*

*I don’t think there’s specific support for that learner because there’s just so many learners … It’s not like you’re going to be like ‘oh she was pregnant, she probably needs a little more attention’. So you know, you won’t get it. So if you survive it, then you’re one of the resilient ones. [Education]*

Others in the education sector indicated that there were counsellors, psychologists and social workers available in some instances:

*Our policy is very clear about … post-delivery. There should be counselling, there should be support measures to support the learner … If the Department of Education cannot assist, we have a referral system whereby we cooperate with the Department of Social Development. [Education]*

*For these girls, I think there should definitely be support for counselling ... by a[n] equipped person. Not by a teacher who’s done a two-month counselling skills course, because that is absolutely nothing. So to have access to a qualified counsellor for that learner. [Health]*

## Discussion

Our previous research with adolescent women, including mothers, living in two low-income peri-urban neighbourhoods found that breastfeeding was seen as important. However, they were unsure how young mothers managed to simultaneously breastfeed and return to school [2].

In this set of interviews, the experiences of health care providers and educators highlighted the disjuncture between broad policy guidance and the lived experience of that policy in local contexts. Our goal, in this discussion, is to highlight and connect the perspectives of health care providers and policy makers, education officials, and local and international policy.

Informants from both sectors were committed to the interests of adolescent mothers and their infants and highlighted the varied ways adolescent mothers and their families managed the learners’ future and infant care. Both health and education informants felt that exclusive breastfeeding for six months was an unrealistic expectation for adolescent mothers but agreed that some period of breast-feeing was desirable. Both health workers and educators agreed that breastfeeding should be promoted, with support offered to adolescents in community settings. Consequently, health worker training around infant feeding must be sufficiently nuanced to address the varied settings of mothers.

Informants recognised that once the learner returned to school, breastfeeding, particularly exclusive breastfeeding, would cease. All agreed that an early return to school would be in the learner-mothers’ interest, and that this needs to be arranged in advance with the girl’s parents. They highlighted learner-mothers’ vulnerability to mental health and academic problems. They perceived that she was dependent on her immediate family, who made decisions about her infant’s care.

Our findings suggest the need for policy that empowers school officials to make choices that better serve the interests of adolescent mothers and their children. Consequently, clear guidelines on the management of scholars late in their pregnancy are required, which together with health services support, would enable school attendance of the pregnant learner until as late in the pregnancy as possible. These guidelines should articulate the necessity for a well-defined recovery and bonding time postpartum, so that educators are in an informed position when discussing return to school with the learner and her parents. Supporting this, findings suggest the opportunity for clinics and community health care workers to assume specific roles supporting exclusive breastfeeding for 4-6 weeks immediately after birth, and thereafter, an important supporting role in correct mixed-feeding (for HIV uninfected women) or formula-feeding. Finally, the importance of active participation of the adolescent mother’s female family members in pregnancy and feeding choices was apparent, highlighting the importance of designing health policies that recognize adolescents’ status as dependents within a household, while supporting them in their role as mothers.

While we focused on policy-level interventions which engage the current reality, we also acknowledge the broader context in which policies are formulated. A longer-term vision is required that engages a broader engagement with the upstream determinants of infant-feeding, and of adolescent pregnancy. This should address issues related to poverty, food security and policy, cultural norms and gender.

Informants’ concerns around school attendance of pregnant learners, particularly during later pregnancy, should be addressed. Continued school attendance is in learners’ interest and should be considered. This implies addressing educators’ fears about health risks, schools’ liability and enlisting health service support.

Historical perspectives for “confinement” of a pregnant woman at around 36 weeks may account for the current tendency to encourage pregnant learners to stay home in the late third trimester. However, health policy does not support this approach. Rather, it may be important to support pregnant adolescents to remain in school for as long as possible. Should the adolescent go into labour, it is unlikely that a primigravida woman with a normal pregnancy would deliver before being transported to a maternity facility.

Education and mental health support for adolescent mothers aged 16-19 seemed to vary and be informal. There should be structured, academic and mental health support that entails public acknowledgement and formalization of policy related to pregnant learners. Mental health screening of pregnant and particularly post-partum learners would be key to detecting mental health disorders, obtaining timeous treatment and support, and reducing the impact of mental health problems on academic outcomes.

There was notable silence around the role (or lack) of fathers, as well as other men, both in our prior research with adolescents, as well as within the education and health sector. This highlights the burden of pregnancy and infant care placed on adolescents and female family members.

### Policy support for exclusive breastfeeding

Policy support for exclusive breastfeeding is strong, given the harmonization of recommendations for six months of exclusive breastfeeding for both HIV infected and uninfected mothers. These recommendations are set against a backdrop of very low rates of exclusive breastfeeding in South Africa [6].

Our study found that key informants from both health and education sectors knew that breastfeeding was important, but the rationale for exclusive breastfeeding was poorly understood. When a full six months of breastfeeding was unfeasible, there was uncertainty and implementers relied on their own understanding as policy was silent. Here more nuanced policy may be beneficial. For example, the benefits of *any* breastfeeding could apply to HIV negative women, in the interest of the infant, and potentially also the mother [20]. In particular, during the six-week postpartum recovery period, active support for breastfeeding may be most valuable, allowing for bonding and for health benefits to be conferred to the baby. Thereafter, recognizing that the return to school will make breastfeeding unlikely, clinics should first support breastfeeding during the period immediately postpartum, followed by supporting a transition to formula or mixed-feeding, depending on the HIV status of the mother. Moreover, health care workers and community health workers should be explicitly educated on formula-feeding [21]. This should include education around safe and correct formula-feeding, managing wasted formula and decision-making about financial resources. Adolescent mothers need hands-on support during the early months postpartum. While our findings show that formula-feeding appears to be the norm, the process of correct, hygienic formula-feeding is not discussed in health settings. Our work suggests that a transition to formula-feeding needs to be recognized and supported.

### Policy that actively supports extended family in their caregiving role

The importance of the infants’ grandmothers in shaping feeding decisions has been previously described in various contexts (Aubel, 2012; Aubel et al., 2001; Bezner Kerr et al., 2008). Most recently, Doherty et al. (2019) highlight the role of grandmothers in shaping feeding decisions in SA, particularly the potential confusion in situations where grandmothers’ feeding recommendations differ from clinic recommendations. Acknowledging the caring role of extended female family members, our research suggests that policy should support direct engagement of extended family throughout the pregnancy and postpartum period.

### Limitations

As a qualitative study with a limited set of questions, it is difficult to interpret silences unless explicitly discussed. Our findings are not necessarily representative of broader experiences. Rather, they demonstrate the ways in which sets of experiences interact and the lived experience of policy. Our work is also geographically specific, and our findings were shaped by the political, educational, and health realities in Cape Town.

## Conclusions

While the global focus on the first 1000 days of life highlights the needs of infants, it acknowledges social determinants of infants’ health, including the well-being of mothers. A mother’s well-being encompasses her ability to feed, nurture and bond with her infant, as well as fulfilling her aspirations to become an autonomous functional adult.

Our study showed that health care providers and policy makers knew that breastfeeding was important, but the motivation for recommending exclusive breastfeeding was less well understood, and therefore less adaptable in cases where a full six months of breastfeeding is impossible. In order to address the needs of adolescent mothers, written policy should speak to the implementers of policy – health care providers and educators, who deal with the needs of learner mothers and their infants. It must not be siloed into health or education services and translate the lived experience of multiple players into implementable guidelines. In this case, where educational and health policy are potentially in conflict, all stakeholders should negotiate on the implementation and translation of lived experience. In particular, breastfeeding policy for school-goers should focus on the period in which breastfeeding is feasible, and actively support a transition to formula-feeding, in order for adolescent mothers to return to school. On the other hand, the educational policy to return to school as soon as possible after birth should not be at the expense of the mother or baby. Rather, defining the potential period of absence may help adolescents to attend school until closer to delivery, and provide a longer period for learners to recover, bond with and breast-feed their child after the birth.

## Supplementary Information

**Additional file 1.**

## Data Availability

The interview guide is added as supplementary material. Recordings and transcripts of interviews are not available as agreed by the Ethics Committee and the study participants as this would violate anonymity and.

## Abbreviations

ARV: Anti-retroviral
HIV: Human Immunodeficiency Virus
LMIC: Low- and middle- income countries
SA: South Africa
UNICEF: United Nations International Children’s Emergency Fund
WHO: World Health Organisation
