# Supplementary material for: Investigating the disjoint between education and health policy for infant feeding among teenage mothers in South Africa: a case for intersectoral work
Source: BMC Public Health. 2022 Jan 6;22:16. doi: 10.1186/s12889-021-12435-8 (PMC8734352; doi:10.1186/s12889-021-12435-8)
Supplement: Supplementary file 1 — Additional file 1. [file 12889_2021_12435_MOESM1_ESM.docx]

## Interview guide for Infant feeding for learner-mothers

Interviewer introduces herself:

1. This research comes from qualitative research done among teenage girls around infant feeding. This is what was said in a focus group:

*I say, do both, breast-feed … get the child used to both bottle-feeding and breast-feeding whilst they are young. So that when you are at home and can breast-feed you are able to and when you are at school you can also leave them with the bottle, because the child will already be used to it. So it’s important to get them used to both while they are still young.*

And another said:

*No I won’t breast-feed, I will leave him with my mother … anyway, so then who is going to continue with the breast-feeding, so I might as well not start.*

What are your thoughts about what was said?

1. **Policy (probe these if not already raised in the discussion above)**

- What do you think of the infant feeding policy – of exclusive breast-feeding for infants until the age of 6 months particularly for adolescent mothers?
  - - What are some of the advantages of the policy?
    - What are some of the obstacles to the policy?

In one focus group the following was said:

*When it comes to school it’s really not easy having a baby and being at school.*

- What do you think of the policy advocating the return of girls post delivery to complete their schooling?
- Do you see any dissonance between these two policies?

2. **Implementation**

- What has been the practice of the infant-feeding policy among teenage girls?
- How do health workers counsel pregnant adolescents?
- What has the education department done at a policy, provincial and school level to support the return to school of adolescents post-partum?

3. **Recommendations**

- What do you think would be viable feeding options for adolescent mothers?
  - What support for counselling?
  - What support for formula if they chose it?
- What would be a viable return to school policy for learner-mothers post-partum?
  - Timing?
  - Support in the school?
    - Counselling
    - Resources: baby at school for 6 months of life?, expressing milk? Home to feed baby?
    - Organisation of mother’s schooling

4. Is there anything else you would like to say about these policies and how they should be implemented across the two sectors?

**Many thanks for your time and reflections**
